# The Relationship Between Hearing Loss and Cognitive Impairment in a Chinese Elderly Population: The Baseline Analysis

**DOI:** 10.3389/fnins.2021.749273

**Published:** 2021-11-26

**Authors:** Xinxing Fu, Bo Liu, Shuo Wang, Robert H. Eikelboom, Dona M. P. Jayakody

**Affiliations:** ^1^Beijing Institute of Otolaryngology, Otolaryngology-Head and Neck Surgery, Beijing Tongren Hospital, Capital Medical University, Beijing, China; ^2^Medical School, The University of Western Australia, Crawley, WA, Australia; ^3^Ear Science Institute Australia, Subiaco, WA, Australia; ^4^Department of Speech Language Pathology and Audiology, University of Pretoria, Pretoria, South Africa; ^5^WA Centre for Health and Ageing, The University of Western Australia, Crawley, WA, Australia

**Keywords:** age-related hearing loss (ARHL), cognitive impairment, depression, loneliness, tonal language

## Abstract

**Objectives:** The objective of the study was to investigate the association between untreated age-related hearing loss and cognitive impairment in Mandarin-speaking older adults living in China.

**Methods:** Older adults (293; 111 males, *M* = 70.33 ± 4.90 years; 182 females, *M* = 69.02 ± 4.08 years) were recruited. All participants completed a pure tone audiometric hearing assessment, Hearing Impairment-Montreal Cognitive Assessment Test (HI-MoCA), and a computerized neuropsychology test battery (CANTAB). The Mandarin version of the De Jong Gierveld Loneliness Scale was used to measure the loneliness, and the Mandarin version of the 21-item Depression Anxiety Stress Scale (DASS-21) was used to measure the current severity of a range of symptoms common to depression, stress, and anxiety of the participants.

**Results:** A multiple stepwise regression analysis showed that the average of four mid-frequency thresholds in the better ear was related to CANTAB Paired Associates Learning (β = 0.20, *p* = 0.002), and the global cognitive function score (HI-MoCA) (β = −0.25, *p* < 0.001). The average of three high frequencies in the better ear was significantly associated with CANTAB Delayed Matching to Sample (β = −0.16, *p* = 0.008), and Spatial Working Memory (β = 0.17, *p* = 0.003).

**Conclusion:** The results revealed a significant relationship between age-related hearing loss and cognitive impairment in Mandarin-speaking older adults. These research outcomes have clinical implications specifically for hearing health care professionals in China and other populations that speak a tonal language, especially when providing hearing rehabilitation.

## Introduction

An estimated 1.57 billion people worldwide had hearing loss in 2019, accounting for 20.3% of the global population (Haile et al., 2021). Approximately one-third of people over 65 years of age are affected by disabling hearing loss. Hearing loss hampers effective communication, which can have a significant impact on the activities of daily life causing feelings of loneliness, isolation, and frustration (Ciorba et al., 2012). Depression, anxiety, and stress are also associated with untreated hearing loss (Jayakody et al., 2018a).

Studies conducted with non-tonal language speakers have established a relationship between untreated age-related hearing loss and cognitive impairment and dementia (Deal et al., 2015; Jayakody et al., 2018b). Those with mild, moderate, and severe hearing loss are two to five times more likely to develop dementia compared with those with normal hearing sensitivity (Lin et al., 2011).

Approximately 50% of the population of the world speaks a tonal language, e.g., Mandarin, Cantonese. In tonal languages, changes in pitch (tone) at the monosyllabic level convey lexical meaning of a word. As pitch is the important factor here, tonal language perception can be equated with music perception. This view is supported by evidence that speaking tonal language leads to enhanced pitch perception in music. The benefits of music training and tonal language experience are bidirectional, and a background in either domain improves processing in the other domain (Ngo et al., 2016). Furthermore, playing a musical instrument may have the benefits on slowing age-related cognitive decline (Balbag et al., 2014). This may be explained by the fact that playing a musical instrument trains executive function involving different cognitive functions of the brain, including memory, visual, and executive functions (Mansky et al., 2020).

There is also psychophysiological evidence that suggests that a tonal language background maybe associated with enhanced general cognitive abilities. A study conducted by using the Corsi blocks tapping test has shown that the Cantonese speakers had a superior working memory related to pitch perception relative to English speakers (Bidelman et al., 2013).

Therefore, it is possible to hypothesize that tonal language speakers are likely to have better working memory in their later life compared with non-tonal language older adults. As hearing sensitivity is strongly associated with age, this raises the question whether speaking a tonal language is a protective factor for cognitive decline in older adults.

There are a handful of studies that have investigated the associations between cognitive function and untreated hearing loss in tonal language speakers. However, these studies have reported conflicting findings. Luo et al. (2018) reported that the prevalence of dementia was 0.61 (95% CI = 0.53–0.71%) with hearing impairment, whereas Gyanwali et al. (2020) failed to find an independent association between hearing loss and dementia (*p* > 0.05).

An analysis of the cognitive assessments utilized in studies that investigated the association between hearing loss and cognitive impairment/dementia in tonal language speakers have not thoroughly assessed cognition, or often used verbally loaded test materials that rely on audition, i.e., some of the materials are presented orally–aurally. For example, a population-based study of age-related hearing loss and dementia in Taiwan was only able to assess the cognitive status of the participants as impaired or normal by psychiatric diagnosis, not enabling a full quantitative analysis (Su et al., 2017). A cohort study investigated the association between hearing loss with cognitive function in a Han Chinese cohort using a standardized neurocognitive battery (Ren et al., 2019). However, as both of these studies used verbal language instructions, it is possible that the hearing loss of the participants had an impact on their cognitive performance, causing an overestimate on the degree of cognition impairment (Dupuis et al., 2015).

The underlying mechanism between hearing loss and cognitive impairment is yet to be established. However, the posited underlying factors of this mechanism may include low education, hypertension, obesity, smoking, depression, physical inactivity, and low social contact (Livingston et al., 2017).

Therefore, this study explored the potential association between hearing loss and cognitive impairment in Mandarin-speaking older adults in China using a non-verbal cognitive assessment test battery, controlling for a number of potential confounders.

## Materials and Methods

Participants were recruited through social media advertisements, flyers, and community centers in Beijing. Ethics approval for this study was obtained from The University of Western Australia—Human Research Ethics Committee (RA/4/20/5538) and Beijing Tongren Hospital, Capital Medical University (TRECKY2019-090). All procedures were carried out in accordance with these approvals, and the participants all provided written informed consent.

Inclusion criteria: Native Mandarin speakers, aged 60 years and above with hearing impairment were invited to participate in the study. Exclusion criteria: Those not in general state of good health or unable to perform tasks required in the cognition evaluation session due to an underlying physical or mental condition, or those who had previously worn or currently wearing hearing aids or a hearing implant were excluded from the study. Those who could not complete the Motor Screening Task (MOT) module of the Cambridge Neuropsychological Test Battery (CANTAB) (Cambridge Cognition Ltd., 2014) due to inability to follow instructions, or dexterity problems were also excluded from the study.

### Measure and Procedure

The assessment methods comprised measurements of hearing ability, cognition function, loneliness, and mental health status.

After enrollment, all participants completed a baseline questionnaire capturing demographic information, and data on alcohol consumption (never, less than 14, 15–28, 29–42, or 43 or more standard drinks per week), smoking (never, past, current, or exposed to second-hand smoking), self-reported chronic disease history (heart disease, stroke, high cholesterol, atherosclerosis, hypertension, diabetes, frequent childhood ear infections, trauma to ear or head, and depression), leisure activities, as well as marital status and living arrangements. Leisure activities were classified into recreational, intellectual, physical, and social categories (Leung et al., 2010). Living arrangements were recorded as living with spouse, living with children, or living alone, and marital status as single, married, widowed, or divorced.

### Hearing Assessment

Pure-tone audiometry (PTA) was assessed with an audiometer (Conera Audiometer, GN Otometrics Ltd., Denmark) and supra-aural earphone (TDH-39). For all participants, bilateral air-conduction thresholds were measured at 0.25, 0.5, 1, 2, 4, 6, and 8 kHz; and bone-conduction thresholds were measured at 0.5, 1, 2, and 4 kHz, through standard audiometric assessment conducted by a qualified audiologist in a soundproof booth at the audiology center of the Beijing Institute of Otolaryngology.

Evaluation of hearing loss was deployed by two different methods: the traditional four frequency average of hearing thresholds at 0.5, 1, 2, and 4 kHz, and the three high frequency average of hearing thresholds at 4, 6, and 8 kHz in the better ear, noted, respectively, as 4FA and 3HFA. These were analyzed as continuous variables. For the purpose of summarizing the hearing loss of the study cohort, the 4FA hearing loss was classified using the World Health Organization (WHO) grades of hearing impairment (Humes, 2019), respectively, normal hearing—less than 20 dB HL; mild hearing loss—20 to < 35 dB HL; moderate hearing loss—35 to < 50 dB HL; moderately severe hearing loss—50 to < 65 dB HL; severe hearing loss—65 to < 80 dB HL; profound hearing loss—80 to < 95 dB HL; complete hearing loss—95 dB HL or greater in the better ear.

### Cognitive Assessment

The global cognitive functions of the subjects were tested using the Montreal Cognitive Assessment Test (MoCA) for the hearing impaired. A Mandarin version of the MoCA (version 7.2) was adapted (Nasreddine et al., 2005), which was converted into a timed PowerPoint (Microsoft Corp., Redmond, Washington, United States) presentation, here named HI-MoCA (Hearing impaired-MoCA), with all the verbal instructions replaced with visual instructions (Lin et al., 2017).

For the visuospatial and executive tasks, an answer sheet was given to the subject to be completed as instructed. For all other tasks, the subjects were asked to verbally answer or respond. For example, for the memory recall section, a series of words were presented at a rate of 2 s per word (truck, banana, violin, desk, and green), comparable to the standard verbal administration of the original MoCA. In the attention tasks, for both the forward and backward order digits repetition, all the numbers were presented at a rate of 1.5 s (compared with one digit per second in the original MoCA). For the digits series task, requiring the participant to identify the digit 1 among a string of digits from 0 to 9 by tapping the table, the HI-MoCA visually presented a series of numbers at a rate of 1.5 s between numbers for the subject to observe and respond to by tapping the table. In the language task, both sentences are presented for 9 s.

All the HI-MoCA tests were administered by an audiologist, who received formal training from a senior neurology physician with advanced clinical experience and completed the certificated training course via the MoCA website.

Non-verbal cognition was assessed using the Cambridge Neuropsychological Test Automated Battery (CANTAB) (Campos-Magdaleno et al., 2020). The CANTAB software was installed on a laptop with an integrated touch screen (IBM, Yoga S, with Windows 10.1 platform).

Evaluation of cognitive function for this study was focused on working and episode memory, processing speed, and spatial information processing. Therefore, the relevant test items were chosen from the CANTAB test battery, including delayed matching to sample (DMS), paired associates learning (PAL), and spatial working memory (SWM), respectively. Assessment commenced with a test of motor function, to qualify the participant for further assessment.

### Motor Screening Task

The motor screening task provides a general assessment of whether vision, sensorimotor deficits, or lack of comprehension, which, if present, would limit the collection of valid data from the participant (Cambridge Cognition Ltd., 2014). It involved the selection of colored crosses in different positions on the screen as quickly and accurately as possible by the participant. MST outcome measures assess the speed of response and the accuracy of pointing of the participant.

### Delayed Matching to Sample

The DMS test assesses both simultaneous visual matching ability and short-term visual recognition memory, for non-verbalizable patterns (Cambridge Cognition Ltd., 2014). DMS is primarily sensitive to damage in the medial temporal lobe area, with some input from the frontal lobes (Olsen et al., 2009). The participant is shown a complex visual pattern (the sample) and four response patterns. The task of the participant is to identify the response pattern that is identical to the sample pattern. Response patterns will be shown simultaneously with the sample pattern, whereas in others, a delay (of 0, 4, or 12 s) is introduced. DMS outcome measures include latency (the speed of response of the participant), the percentage of correct patterns selected, and a statistical measure giving the probability of an error after a correct or incorrect response. The DMS parallel mode was administered for all the subjects, including three practice trials and 20 assessment trials. The average administration time is around 8 min. Percentage of correct patterns selected by the participant was analyzed in this study (Cambridge Cognition Ltd., 2014).

### Paired Associates Learning

The PAL test assesses episodic visuospatial memory, learning, and association ability (Cambridge Cognition Ltd., 2014). This test is primarily sensitive to changes in medial temporal lobe functioning (Cambridge Cognition Ltd., 2014). In this task, six to eight white boxes are displayed on a computer screen. These briefly reveal a pattern which varies in shape and color. The task of the participant is to remember the pattern revealed and match it to the pattern that appears in the middle of the screen by touching the box that contains the correct pattern. The task is made progressively difficult by presenting 1, 2, 3, 6, and 8 patterns to the participant. Outcome measures include the errors made by the participant, the number of trials required to locate the pattern(s) correctly, memory scores, and stages completed. The PAL clinical mode was administered for all the subjects with up to eight stages depending on the performance of the subjects; each stage may have up to 10 trials (attempts) in total. The average administration time is around 10 min but is greatly affected by the degree of impairment of the subject and the number of repeat presentations that are required. PAL errors (total shapes) and PAL errors (six shapes) were analyzed in this study (Cambridge Cognition Ltd., 2014).

### Spatial Working Memory

The SWM test requires retention and manipulation of visuospatial information. This self-ordered test has obvious executive function demands and provides a measure of non-verbal working memory, visuospatial working memory, and strategy use (Cambridge Cognition Ltd., 2014). SWM is a sensitive measure of frontal lobe and “executive” dysfunction. The task of the participant was to locate tokens hidden in increasing number of boxes (3, 4, 6, and 8). Each box contained only one token per sequence. Searching a box more than once during a sequence results in a “within search error” and revisiting a box in which a token has been found before incurs a “between search error.” A “strategy” score, calculated for the more difficult six- and eight-box levels, represented the use of an efficient strategy based on predetermined sequence. Poor use of strategy is reflected in higher strategy scores and vice versa. Three main outcome measures, spatial working memory within errors, between errors, and strategy scores, were calculated for the purpose of this study. The SWM clinical mode was administered for all the subjects with 16 stages, including four practices and 12 assessment trials. The average administration time is around 9 min but is also affected by the degree of impairment of the subjects.

### Loneliness Measurement

The Mandarin version of the six-item De Jong Gierveld Loneliness Scale was used to measure the loneliness of the subjects (Leung et al., 2008; Yang et al., 2018; Fung et al., 2019). In this six-item scale, three statements are made about emotional loneliness and three about social loneliness. There are negatively (items 1, 2, and 3) and positively (items 4, 5, and 6) worded items, scored 1 point for “Yes” and “More or less” answers, and 0 points for “No” answers to questions 1–3; scored 1 point for “More or less” and “No” answers, and 0 points for “Yes” answers to questions 4–6.

### Assessment of Depression, Anxiety, and Stress

The Depression Anxiety Stress Scales (DASS-21) is a questionnaire to assess the symptoms of depression, anxiety, and stress (Chan et al., 2012; Wang K. et al., 2016). The 21 items on the questionnaire comprise a set of three self-reported scales designed to assess depression, anxiety, and stress status of subjects. The seven elements on each scale are graded on a Likert scale from 0 to 3 (0: “Did not apply to me at all,” 1: “Applied to me to some degree or some of the time,” 2: “Applied to me to a considerable degree or a good part of the time,” and 3: “Applied to me very much or most of the time”). Depression, anxiety, and stress scores are determined by summarizing the scores of the related items (Lovibond and Lovibond, 1995). As the DASS-21 is a shorter version of the 42-item original DASS, the score for each subscale is multiplied by two to calculate the final score.

### Statistics Analysis

All statistical analyses were performed using SPSS version 25 (SPSS Inc., Chicago, IL, United States). A multiple forward stepwise regression analysis was used to examine the relationship between cognitive functions and other variables, including age, gender, smoking and alcohol consumption, chronic medical history, education years, loneliness and mental health, and PTA thresholds of better ear average across 500 Hz–8 kHz. The different CANTAB modules and HI-MoCA scores were entered as dependent variables, respectively. The collinearity tests were examined during each stepwise regression analysis.

At each step of the regression analysis, variables were added based on *p*-values, and the Akaike Information Criterion (AIC) was used to set a limit on the total number of variables included in the final model. The criteria of entry of each variable was a probability of *F* ≤ 0.05 and removal of each variable a probability of *F* ≥ 0.10.

## Results

### Descriptive Data

The demographic information, cognitive function scores, and other descriptive data of this study population are presented in Table 1, including the age, gender, education years, hearing threshold, smoking status, alcohol intake, history of chronic disease, depression, anxiety, and stress status of the participants. A total of 293 Mandarin speakers aged between 60 and 87 years were included in the study. The study cohort consisted of 111 males (mean age = 70.33 ± 4.90 years) and 182 females (mean age = 69.02 ± 4.08 years).

**TABLE 1:** Descriptive data of the participants.

Characteristics	All subjects
	*N* = 293
Male sex, no. (%)	111 (37.9%)
Smoking, no. (%)	107 (36.5%)
Alcohol consumption^a^, no. (%)	279 (95.2%)
Vascular disease^b^, no. (%)	231 (78.8%)
Diabetes, no. (%)	66 (22.5%)
Frequent childhood ear infections, no. (%)	13 (4.4%)
Depression clinically diagnosed, no. (%)	10 (3.4)
Self-report HL, no. (%)	212 (72.4%)
Noise exposure, no. (%)	52 (17.7%)
Living alone, no. (%)	17 (5.8%)
Single or divorced, no. (%)	33 (11.3%)
Age (years)	69.52 ± 4.45
Education (years)	12.98 ± 2.84
Hearing loss classification, no. (%)	
Normal hearing	65 (22.2%)
Mild hearing loss	106 (36.2%)
Moderate hearing loss	54 (18.4%)
Moderately severe hearing loss	34 (11.6%)
Severe hearing loss	31 (10.6%)
Profound hearing loss	3 (1.0%)
4FA (dB HL)	36.94 ± 18.78
3HFA (dB HL)	52.31 ± 20.17
Emotional loneliness	0.92 ± 0.84
Social loneliness	1.11 ± 1.18
Loneliness	2.03 ± 1.59
Depression	3.65 ± 5.22
Anxiety	5.98 ± 5.58
Stress	6.45 ± 6.70
Recreational activities score	14.5 ± 10.68
Intellectual activities score	20.87 ± 12.72
Physical activities score	21.15 ± 11.06
Social activities score	2.11 ± 2.79
HI-MoCA score	25.00 ± 2.63
SWM between error	35.99 ± 16.25
SWM within error	6.83 ± 5.49
SWM strategy	34.64 ± 5.21
PAL error (all shapes)	32.54 ± 26.66
PAL error (six shapes)	7.60 ± 7.66
DMS correct percent	79.43 ± 11.75

*The data are presented as means ± standard deviations, unless otherwise indicated.*

*4FA: four frequencies (500 Hz, 1, 2, and 4 kHz) average of pure tone hearing thresholds of the better ear; 3HFA: three high frequencies (4, 6, and 8 kHz) average of pure tone hearing thresholds of the better ear; HI-MoCA, hearing impaired-Montreal cognitive assessment test; PAL, paired associates learning; DMS, delayed matching to sample; SWM, spatial working memory.*

*^a^Alcohol consumption reference is never or less than 14 standard drinks per week.*

*^b^Vascular disease indicates any one of heart disease, stroke, high cholesterol, atherosclerosis, or hypertension.*

For descriptive purposes, the better ear 4FA was used to group the participants; 65 had normal hearing, 106 had mild hearing loss, 54 had moderate hearing loss, 34 had moderately severe hearing loss, 31 had severe hearing loss, and three had profound hearing loss, as shown in Table 1.

### Regression Analysis

The multiple stepwise regression analyses revealed that the better ear 4FA significantly predicted HI-MoCA scores (*p* < 0.001), PAL error for 6 shapes (*p* < 0.05), and that the better ear 3HFA significantly predicted SWM within error (*p* < 0.05) and DMS correct percent (*p* < 0.05). The detailed results are shown in Table 2. Only the last model of the stepwise regression is shown. Each model of forward stepwise regression for HI-MoCA, SWM between error, SWM within error, SWM strategy, PAL error (all shapes), PAL error (six shapes), DMS correct percent are, respectively, shown in Supplementary Tables 1–7.

**TABLE 2:** Multiple stepwise regression between CANTAB/HI-MoCA scores and other variables.

Dependent variable	Independent variables	*R* ^2^	Adjusted *R*^2^	B	β	*t*	Sig.	95% Confidence interval for B	
								Lower bound	Upper bound
HI-MoCA score	4FA	0.09	0.08	−0.04	−0.25	−4.03	0.000	−0.05	−0.02
	Self-report HL			1.04	0.18	2.88	0.004	0.33	1.75
	Education years			0.14	0.15	2.63	0.009	0.04	0.24
SWM between error	Age	0.09	0.08	0.76	0.21	3.69	0.000	0.36	1.17
	Social loneliness			−5.24	−0.38	−3.46	0.001	−8.22	−2.26
	Loneliness			2.69	0.26	2.41	0.017	0.49	4.89
SWM within error	3HFA	0.04	0.04	0.05	0.17	2.99	0.003	0.02	0.08
	Education years			0.26	0.14	2.34	0.020	0.04	0.48
SWM strategy	Social loneliness	0.06	0.05	−2.02	−0.46	−3.99	0.000	−3.01	−1.02
	Loneliness			1.19	0.36	3.11	0.002	0.44	1.94
	Anxiety			−0.13	−0.14	−2.29	0.023	−0.24	−0.02
PAL error (All shapes)	Education years	0.11	0.09	−1.58	−0.17	−2.95	0.003	−2.63	−0.53
	Intelligent activities			−0.35	−0.16	−2.89	0.004	−0.58	−0.11
	Gender			8.93	0.16	2.82	0.005	2.70	15.15
	Social activities			1.37	0.14	2.52	0.012	0.30	2.45
	Living arrangements			14.96	0.13	2.32	0.021	2.25	27.67
PAL error (6 shapes)	4FA	0.08	0.07	0.08	0.20	3.16	0.002	0.03	0.13
	Self-report HL			−2.78	−0.16	−2.64	0.009	−4.86	−0.71
	Depression			0.19	0.13	2.31	0.021	0.03	0.36
	Education years			−0.32	−0.12	−2.09	0.038	−0.63	−0.02
DMS correct percent	3HFA	0.12	0.10	−0.09	−0.16	−2.66	0.008	−0.16	−0.02
	Education years			0.57	0.14	2.43	0.016	0.11	1.03
	Age			−0.31	−0.12	−2.02	0.045	−0.62	−0.01
	Anxiety			−0.26	−0.12	−2.22	0.027	−0.49	−0.03
	Gender			−2.88	−0.12	−2.06	0.040	−5.64	−0.13

*4FA, four frequencies (500 Hz, 1, 2, and 4 kHz) average of pure-tone hearing thresholds of the better ear; HI-MoCA, hearing impaired-Montreal cognitive assessment; PAL, paired associates learning; DMS, delayed matching to sample; SWM, spatial working memory. 3HFA, three high frequencies (4, 6, and 8 kHz) average of pure-tone hearing thresholds of the better ear.*

## Discussion

This study investigated the association between hearing loss and global cognitive abilities (HI-MoCA) and multiple domains of non-verbal cognitive abilities in Mandarin-speaking older adults. To the best of our knowledge, this is the first study to elucidate the association between hearing loss and cognitive function by using the hearing impairment version MoCA and a non-verbal neurocognitive assessment tool with tonal language participants.

Our results are consistent with previous cross-sectional studies of pure-tone audiometric hearing loss and cognitive decline and impairment in non-tonal language speakers (Gussekloo et al., 2005; Deal et al., 2015; Jayakody et al., 2018b). Our results revealed that both mid-frequency hearing sensitivity and self-reported hearing loss were significantly associated with HI-MoCA score.

As shown in Table 2, self-reported hearing loss was significantly associated with both HI-MoCA (β = 0.18, *p* = 0.004) and PAL error (six shapes) (β = −0.16, *p* = 0.009). This finding suggests that self-reported hearing loss may be a useful indicator when evaluating the relationship between hearing loss and cognitive impairment. This finding accords with another study, which showed that self-reported hearing loss was significantly associated with both baseline and follow-up cognitive impairment (Amieva et al., 2015).

Our results also showed that the age-related hearing loss significantly associated with poor performance on some of the cognitive domains, specifically DMS (short-term visual memory) and PAL (episodic visuospatial memory). Prior studies have noted that episodic memory impairments are one of the earliest signs of amnestic mild cognitive impairment, Alzheimer’s disease, and other dementias (Wang P. et al., 2016).

Short-term visual memory function in this study was assessed using DMS module of the CANTAB test battery. Linear regression showed that while high-frequency hearing in the better ear was significantly associated with the DMS score, mid-frequency hearing was not associated with the DMS score. This finding is consistent with previous research (Jayakody et al., 2018b) on non-tonal language speakers. A possible explanation for this is that presbycusis usually begins with reduced hearing sensitivity in the higher frequencies while remaining relatively better in the low and mid-frequency range. Consequently, the association between DMS and mid-frequency hearing impairment was not observed in the regression analysis.

Visual memory and learning ability were measured by PAL task of CANTAB test battery. There was a significant positive correlation between mid-frequency hearing in (4FA) in the better ear, and PAL error (six shapes). Previous studies have also demonstrated that performance on the PAL task is highly associated with cognition impairment, and can differentiate subjects with Alzheimer’s disease from normal controls with high sensitivity and specificity (Hicks et al., 2020). However, no significant association was found between PAL scores (all shapes) and hearing loss.

Two types of leisure activities (intelligent and social) were observed in the regression model to be associated with PAL error (all shapes), but not PAL error (6 shapes). Prior studies that have noted the importance of participation in leisure activities, which has been associated with the reduced risk of cognitive impairment after controlling for age, gender, and education (Verghese et al., 2003). In this study, information regarding four categories of leisure activities was collected by a questionnaire. It is difficult to explain this result that why PAL error (all shapes) and PAL error (six shapes) showed different regression outcomes, but it may be related to the high level of difficultly of the PAL eight shapes task. Participants sometimes took a very long time on the task and lost their patience, and the results are likely to be inaccurate and unreliable.

Spatial working memory requires retention and manipulation of visuospatial information. The SWM task has notable demands on executive function and provides a measure of strategy as well as working memory. This study showed that high frequency hearing is significantly associated with SWM within error, but not with mid-frequency hearing. This result may be explained, as noted earlier, by the fact that high frequencies show a decrease in sensitivity at least a decade earlier than for the mid-frequency speech frequencies (Salvi et al., 2018). However, neither 4HA nor 3HFA were associated with SWM between error and SWM strategy. This finding is contrary to a previous study which has suggested that both the SWM errors and strategy score are associated with mid-frequency hearing (Jayakody et al., 2018b).

Education was significantly associated with almost all the cognitive-dependent variables, including HI-MoCA and CANTAB DMS, PAL, and SWM. These results corroborate the findings of a great deal of the previous work in this field. Lower rates of late-life dementia are associated with higher education levels during earlier life (Livingston et al., 2017). Early education may promote the development during a sensitive period of childhood that protect against late-life cognitive decline (Zahodne et al., 2015).

Another important finding was that social loneliness and overall loneliness (social and emotional) were significantly associated with both SWM between error and SWM strategy. Several reports have shown that both loneliness and social isolation are associated with cognitive decline (Donovan et al., 2017; Lara et al., 2019). The literature does not testify the causal relationship between loneliness and cognitive function. Loneliness may lead to cognitive decline, perhaps from limited social interactions, but it is also possible that cognitive impairment may lead to limited social interactions and further loneliness (Boss et al., 2015).

Between 20 and 40% of the elderly in Western countries are reported to be lonely (Savikko et al., 2005), and the prevalence of loneliness has increased from 16 to 30% from 1992 to 2000 (Yang and Victor, 2008), possibly due to the impact of the “empty-nester” phenomenon that had impact on that (Fung et al., 2019). However, among the participants in this study, only 5.8% lived alone, the majority living with their children and even grandchildren. In China, elderly people tend to live with their children, playing an important role in caring for their grandchildren. This living arrangement is likely to have an impact on mental wellbeing and cognitive functioning (Mazzuco et al., 2017), as well as possible decreasing of loneliness and social isolation. This contrasts with the prevailing culture in Western countries, where parents tend to live alone after children move out of home, and they tend to live in residential aged-care facilities later in life; however, less than 2% of the subjects of this study population lived in aged-care centers.

### Clinical Implications

This study contributes evidence to the growing global body of evidence that hearing loss and cognitive decline are significantly associated, also in Mandarin-speaking older adults. While the risk of hearing loss to cognition has not been determined, the association is something that hearing health professionals need to bear in mind when managing and counseling their patients.

### Limitations

One of the limitations in this study is that the age of participants recruited generally reflected their level of hearing loss and that there were relatively fewer participants with increasing severity of hearing loss. However, this is consistent with the prevalence of hearing loss in the population in relation to age. This study was designed as a longitudinal study, so as to examine the association between ARHL and cognitive decline over time. Another potential limitation is that participant fatigue may have affected some of the more strenuous tests.

### Future Directions

These findings warrant longitudinal studies to better understand the association between hearing loss and cognitive impairment over time and also to investigate how confounding factors contribute to the cognitive functions overall. Recent studies have studied the impact of hearing aids and cochlear implantation on cognitive functions of older adults with hearing loss (Jayakody et al., 2017, 2020; Kramer et al., 2018; Maharani et al., 2018). The results indicate that hearing rehabilitation has some degree of impact on improving cognitive functions. Based on the findings, we recommend a randomized control trial investigating the impact of hearing rehabilitation on the cognitive functions of Mandarin-speaking older adults.

## Conclusion

This study provides more evidence for the association between hearing loss and cognitive impairment in Mandarin-speaking older adults. This study revealed that the hearing loss has a significant relationship with cognitive impairment in a Chinese-speaking population.

## Data Availability Statement

The original contributions presented in the study are included in the article/Supplementary Material, further inquiries can be directed to the corresponding author/s.

## Ethics Statement

The studies involving human participants were reviewed and approved by the University of Western Australia-Human Research Ethics Committee, the Institutional Review Board of Beijing Tongren Hospital, Capital Medical University. The participants provided their written informed consent to participate in this study.

## Author Contributions

DJ and XF designed the experiments. XF carried out the experiments and wrote the manuscript. XF, BL, SW, RE, and DJ analyzed the experimental results. RE, DJ, BL, and SW reviewed the manuscript. All authors contributed to the article and approved the submitted version.

## Conflict of Interest

The authors declare that the research was conducted in the absence of any commercial or financial relationships that could be construed as a potential conflict of interest. The editor KL has declared a shared institutional affiliation with the authors XF, BL, and SW at the time of review.

## Publisher’s Note

All claims expressed in this article are solely those of the authors and do not necessarily represent those of their affiliated organizations, or those of the publisher, the editors and the reviewers. Any product that may be evaluated in this article, or claim that may be made by its manufacturer, is not guaranteed or endorsed by the publisher.
