# Supplementary material for: The Relationship Between Hearing Loss and Cognitive Impairment in a Chinese Elderly Population: The Baseline Analysis
Source: Front Neurosci. 2021 Nov 26;15:749273. doi: 10.3389/fnins.2021.749273 (PMC8662817; doi:10.3389/fnins.2021.749273)
Supplement: Supplementary file 1 [file Data_Sheet_1.docx]

Supplementary material

Table S-1: Multiple stepwise regression between HI-MoCA scores and other variables.

|  | Model 1 | | | | Model 2 | | | | Model 3 | | | |
| --- | --- | --- | --- | --- | --- | --- | --- | --- | --- | --- | --- | --- |
| Variables | B | SE | β | Sig. | B | SE | β | Sig. | B | SE | β | Sig. |
| 4FA | -0.03 | 0.01 | -0.20 | 0.001 | -0.04 | 0.01 | -0.27 | 0.000 | -0.03 | 0.01 | -0.25 | 0.000 |
| Self-Report HL |  |  |  |  | 1.03 | 0.36 | 0.17 | 0.005 | 1.04 | 0.36 | 0.18 | 0.004 |
| Education Years |  |  |  |  |  |  |  |  | 0.14 | 0.05 | 0.15 | 0.009 |
| R^2^ | 0.040 | | |  | 0.065 | | |  | 0.087 | | |  |
| Std. Error of the Estimate | 2.586 | | |  | 2.556 | | |  | 2.530 | | |  |

Table S-2: Multiple stepwise regression between SWM between error scores and other variables.

|  | Model 1 | | | | Model 2 | | | | Model 3 | | | |
| --- | --- | --- | --- | --- | --- | --- | --- | --- | --- | --- | --- | --- |
| Variables | B | SE | β | Sig. | B | SE | β | Sig. | B | SE | β | Sig. |
| Age | 0.83 | 0.21 | 0.23 | 0.000 | 0.79 | 0.21 | 0.22 | 0.000 | 0.76 | 0.21 | 0.21 | 0.000 |
| Social Loneliness |  |  |  |  | -2.10 | 0.78 | -0.15 | 0.008 | -5.24 | 1.52 | -0.38 | 0.001 |
| Loneliness |  |  |  |  |  |  |  |  | 2.69 | 1.12 | 0.26 | 0.017 |
| R^2^ | 0.052 | | |  | 0.075 | | |  | 0.093 | | |  |
| Std. Error of the Estimate | 15.852 | | |  | 15.685 | | |  | 15.556 | | |  |

Table S-3: Multiple stepwise regression between SWM within error scores and other variables.

|  | Model 1 | | | | Model 2 | | | |
| --- | --- | --- | --- | --- | --- | --- | --- | --- |
| Variables | B | SE | β | Sig. | B | SE | β | Sig. |
| 3HFA | 0.04 | 0.02 | 0.16 | 0.007 | 0.05 | 0.02 | 0.17 | 0.003 |
| Education Years |  |  |  |  | 0.26 | 0.11 | 0.14 | 0.020 |
| R^2^ | 0.025 | | |  | 0.043 | | |  |
| Std. Error of the Estimate | 5.433 | | |  | 5.391 | | |  |

Table S-4: Multiple stepwise regression between SWM strategy scores and other variables.

|  | Model 1 | | | | Model 2 | | | | Model 3 | | | |
| --- | --- | --- | --- | --- | --- | --- | --- | --- | --- | --- | --- | --- |
| Variables | B | SE | β | Sig. | B | SE | β | Sig. | B | SE | β | Sig. |
| Social Loneliness | -0.68 | 0.26 | -0.15 | 0.008 | -1.76 | 0.50 | -0.40 | 0.000 | -2.02 | 0.51 | -0.46 | 0.000 |
| Loneliness |  |  |  |  | 0.93 | 0.37 | 0.28 | 0.012 | 1.19 | 0.38 | 0.36 | 0.002 |
| Anxiety |  |  |  |  |  |  |  |  | -0.13 | 0.06 | -0.14 | 0.023 |
| R^2^ | 0.024 | | |  | 0.038 | | |  | 0.052 | | |  |
| Std. Error of the Estimate | 5.157 | | |  | 5.110 | | |  | 5.073 | | |  |

Table S-5: Multiple stepwise regression between PAL error (All shapes) scores and other variables.

|  | Model 1 | | | | Model 2 | | | | Model 3 | | | | Model 4 | | | | Model 5 | | | |
| --- | --- | --- | --- | --- | --- | --- | --- | --- | --- | --- | --- | --- | --- | --- | --- | --- | --- | --- | --- | --- |
| Variables | B | SE | β | Sig. | B | SE | β | Sig. | B | SE | β | Sig. | B | SE | β | Sig. | B | SE | β | Sig. |
| Education Years | -1.82 | 0.54 | -0.19 | 0.001 | -1.59 | 0.54 | -0.17 | 0.004 | -1.49 | 0.54 | -0.16 | 0.006 | -1.59 | 0.54 | -0.17 | 0.003 | -1.58 | 0.53 | -0.17 | 0.003 |
| Intelligent Activities |  |  |  |  | -0.32 | 0.12 | -0.15 | 0.009 | -0.30 | 0.12 | -0.14 | 0.012 | -0.33 | 0.12 | -0.16 | 0.006 | -0.35 | 0.12 | -0.16 | 0.004 |
| Gender |  |  |  |  |  |  |  |  | 6.58 | 3.13 | 0.12 | 0.036 | 7.69 | 3.14 | 0.14 | 0.015 | 8.93 | 3.16 | 0.16 | 0.005 |
| Social Activities |  |  |  |  |  |  |  |  |  |  |  |  | 1.29 | 0.55 | 0.14 | 0.019 | 1.37 | 0.55 | 0.14 | 0.012 |
| Living arrangements |  |  |  |  |  |  |  |  |  |  |  |  |  |  |  |  | 14.96 | 6.46 | 0.13 | 0.021 |
| R^2^ | 0.038 | | |  | 0.054 | | |  | 0.065 | | |  | 0.079 | | | | 0.093 | | | |
| Std. Error of the Estimate | 26.199 | | |  | 25.935 | | |  | 25.783 | | |  | 25.582 | | | | 25.390 | | | |

Table S-6: Multiple stepwise regression between PAL error (6 shapes) scores and other variables.

|  | Model 1 | | | | Model 2 | | | | Model 3 | | | | Model 4 | | | |
| --- | --- | --- | --- | --- | --- | --- | --- | --- | --- | --- | --- | --- | --- | --- | --- | --- |
| Variables | B | SE | β | Sig. | B | SE | β | Sig. | B | SE | β | Sig. | B | SE | β | Sig. |
| 4FA | 0.06 | 0.02 | 0.15 | 0.008 | 0.09 | 0.03 | 0.22 | 0.001 | 0.09 | 0.03 | 0.21 | 0.001 | 0.08 | 0.03 | 0.20 | 0.002 |
| Self-Report HL |  |  |  |  | -2.80 | 1.07 | -0.16 | 0.009 | -2.76 | 1.06 | -0.16 | 0.010 | -2.78 | 1.06 | -0.16 | 0.009 |
| Depression |  |  |  |  |  |  |  |  | 0.21 | 0.08 | 0.14 | 0.014 | 0.19 | 0.08 | 0.13 | 0.021 |
| Education Years |  |  |  |  |  |  |  |  |  |  |  |  | -0.32 | 0.15 | -0.12 | 0.038 |
| R^2^ | 0.021 | | |  | 0.040 | | |  | 0.057 | | |  | 0.068 | | | |
| Std. Error of the Estimate | 7.579 | | |  | 7.504 | | |  | 7.438 | | |  | 7.395 | | | |

Table S-7: Multiple stepwise regression between DMS correct percent scores and other variables.

|  | Model 1 | | | | Model 2 | | | | Model 3 | | | | Model 4 | | | | Model 5 | | | |
| --- | --- | --- | --- | --- | --- | --- | --- | --- | --- | --- | --- | --- | --- | --- | --- | --- | --- | --- | --- | --- |
| Variables | B | SE | β | Sig. | B | SE | β | Sig. | B | SE | β | Sig. | B | SE | β | Sig. | B | SE | β | Sig. |
| 3HFA | -0.14 | 0.03 | -0.24 | 0.000 | -0.13 | 0.03 | -0.22 | 0.000 | -0.11 | 0.03 | -0.18 | 0.002 | -0.11 | 0.03 | -0.18 | 0.002 | 0.57 | 0.23 | 0.14 | 0.008 |
| Education Years |  |  |  |  | 0.57 | 0.24 | 0.14 | 0.016 | 0.62 | 0.24 | 0.15 | 0.009 | 0.61 | 0.23 | 0.15 | 0.010 | -0.31 | 0.15 | -0.12 | 0.016 |
| Age |  |  |  |  |  |  |  |  | -0.34 | 0.16 | -0.13 | 0.031 | -0.34 | 0.15 | -0.13 | 0.031 | -0.26 | 0.12 | -0.12 | 0.045 |
| Anxiety |  |  |  |  |  |  |  |  |  |  |  |  | -0.24 | 0.12 | -0.11 | 0.041 | -2.88 | 1.40 | -0.12 | 0.027 |
| Gender |  |  |  |  |  |  |  |  |  |  |  |  |  |  |  |  | 0.57 | 0.23 | 0.14 | 0.040 |
| R^2^ | 0.052 | | |  | 0.068 | | |  | 0.080 | | |  | 0.090 | | | | 0.100 | | | |
| Std. Error of the Estimate | 11.441 | | |  | 11.345 | | |  | 11.274 | | |  | 11.211 | | | | 11.149 | | | |

SE: Std. Error.

4FA: four frequencies (500, 1 kHz, 2 kHz and 4 kHz) average of pure tone hearing thresholds of the better ear; HI-MoCA: hearing impaired-Montreal cognitive assessment; PAL: paired associates learning; DMS: delayed matching to sample; SWM: spatial working memory. 3HFA: three high frequencies (4,6 kHz and 8 kHz) average of pure tone hearing thresholds of the better ear.
